# Clinical Course, Myopathology and Challenge of Therapeutic Intervention in Pediatric Patients with Autoimmune-Mediated Necrotizing Myopathy

**DOI:** 10.3390/children8090721

**Published:** 2021-08-24

**Authors:** Adela Della Marina, Marc Pawlitzki, Tobias Ruck, Andreas van Baalen, Nadine Vogt, Bernd Schweiger, Swantje Hertel, Heike Kölbel, Heinz Wiendl, Corinna Preuße, Andreas Roos, Ulrike Schara-Schmidt

**Affiliations:** 1 Centre for Neuromuscular Disorders and Centre for Translational Neuro- and Behavioral Sciences, Department of Pediatric Neurology University Hospital Essen, University Duisburg-Essen, 45147 Essen, Germany; Swantje.Hertel@uk-essen.de (S.H.); Heike.Koelbel@uk-essen.de (H.K.); Andreas.Roos@uk-essen.de (A.R.); Ulrike.Schara-Schmidt@uk-essen.de (U.S.-S.); 2 Department of Child and Adolescent Psychiatry and Psychotherapy, University Hospital Münster, 48149 Münster, Germany; marc.pawlitzki@ukmuenster.de; 3 Department of Neurology with Institute of Translational Neurology, University Hospital Münster, 48149 Münster, Germany; tobias.ruck@med.uni-duesseldorf.de (T.R.); heinz.wiendl@ukmuenster.de (H.W.); 4 Department of Neurology, University Hospital Düsseldorf, Heinrich-Heine-University Düsseldorf, 40225 Düsseldorf, Germany; 5 Clinic for Child and Adolescent Medicine II, University Hospital Schleswig-Holstein, 24105 Kiel, Germany; van.baalen@pedneuro.uni-kiel.de (A.v.B.); Nadine.Vogt@uksh.de (N.V.); 6 Institute of Diagnostic and Interventional Radiology and Neuroradiology, University Hospital Essen, University Duisburg-Essen, 45147 Essen, Germany; bernd.schweiger@uk-essen.de; 7 Department of Neuropathology, Humboldt-Universität zu Berlin and Berlin Institute of Health, Charité-Universitätsmedizin Berlin, 10117 Berlin, Germany; corinna.preusse@charite.de; 8 Children’s Hospital of Eastern Ontario Research Institute, University of Ottawa, Ottawa, ON K1H 8L1, Canada

**Keywords:** signal recognition particle, 3-hydroxy-3-methylglutaryl, coenzyme A reductase, juvenile myositis, therapy, clinical course, chaperone-assisted autophagy

## Abstract

(1) Background: Immune–mediated necrotizing myopathy (IMNM) is a rare form of inflammatory muscle disease which is even more rare in pediatric patients. To increase the knowledge of juvenile IMNM, we here present the clinical findings on long-term follow-up, myopathological changes, and therapeutic strategies in two juvenile patients. (2) Methods: Investigations included phenotyping, determination of antibody status, microscopy on muscle biopsies, MRI, and response to therapeutic interventions. (3) Results: Anti-signal recognition particle (anti-SRP54) and anti- 3-hydroxy-3-methylglutarly coenzyme A reductase (anti-HMGCR) antibodies (Ab) were detected in the patients. Limb girdle presentation, very high CK-levels, and a lack of skin rash at disease-manifestation and an absence of prominent inflammatory signs accompanied by an abnormal distribution of α-dystroglycan in muscle biopsies initially hinted toward a genetically caused muscle dystrophy. Further immunostaining studies revealed an increase of proteins involved in chaperone-assisted autophagy (CASA), a finding already described in adult IMNM-patients. Asymmetrical muscular weakness was present in the anti-SRP54 positive Ab patient. After initial stabilization under therapy with intravenous immunoglobulins and methotrexate, both patients experienced a worsening of their symptoms and despite further therapy escalation, developed a permanent reduction of their muscle strength and muscular atrophy. (4) Conclusions: Diagnosis of juvenile IMNM might be complicated by asymmetric muscle weakness, lack of cutaneous features, absence of prominent inflammatory changes in the biopsy, and altered α-dystroglycan.

## 1. Introduction

Autoimmune-mediated necrotizing myopathy (IMNM) is a rare subgroup of idiopatic inflammatory myopathies (IIM) and associated with the anti-signal-recognition particle (anti-SRP54) or the anti–3-hydroxy-3-methylglutaryl-coenzyme A reductase (anti-HMGCR) autoantibodies (Ab) [1,2]. Juvenile dermatomyositis (JDM) is the most common IIM in children, with an incidence of 3.2 per million children [3], positivity for anti-HMGCR and anti-SRP54 Ab were present in only 1–2.2 % of pediatric series from the UK and the USA with IIM [1,2,4,5]. In a cohort of 387 pediatric and adult patients from Japan with IIM, 18% were anti-SRP54 and 12% were anti-HMGCR Ab positive [6], and 5% of those patients had an onset of their symptoms before 18 years of age. The presence of muscle cell necrosis and muscle cell regeneration are the histopathological hallmarks in IMNM-patient derived muscle biopsies, inflammatory cells are sparse or only slightly localized in the perivascular compartment [7,8]. Disease activity is almost always associated with elevated creatine kinase (CK) levels and subacute symmetrical proximal muscular weakness is present in all patients [9]. The typical skin rash as in JDM is less common and due to the slowly progressive course in some patients and the development of muscle atrophy,limb-girdle muscular dystrophy may be presumed [10]. Pathophysiologically, perturbed proteostasis accompanied by an increase of sarcoplasmic chaperones, lysosomal proteins, and aggregation markers was described independently of the antibody status [11,12].

To date, no standardized therapy procedures exist, and some expert recommendations suggest that rituximab should be used in anti-SRP54 and anti-HMGCR Ab positive patients who fail to respond to steroids and intravenous immunoglobulins (IVIG) as second line treatments [13,14]. Patients with anti-SRP54 Ab tend to have a more severe disease course compared to anti-HMGCR Ab positive patients. Younger age at disease onset is associated with more severe symptoms that can be resistant to treatment and therefore poorer prognosis [6,15]. Recent case series in children with anti-SRP54 Ab implicate that an early and intensive combination of immunosuppressive therapy and physiotherapy may lead to early stabilization of the disease and better outcome, although long-term observational studies are still lacking [16]. Similar escalation in therapy was applied in juvenile patients with anti-HMGCR Ab myopathy in cases of severe weakness [2,5], but in some mild-affected cases, IVIG monotherapy led to remission and normalization of CK-levels [10].

Here, we describe the clinical and myopathological findings as well as the therapeutic challenges of two pediatric patients with IMNM on long-term follow up. In both, due to profound muscle weakness and the undulating course of the disease, it was difficult to determine the most suitable moment to adjust, terminate, or escalate the respective therapies. Of note, in the long term, both patients developed muscular atrophy and persisting muscular weakness.

## 2. Materials and Methods

### 2.1. Patient Recruitment

We recruited one patient from the Neuromuscular Center, Department of Child Neurology, Children’s Hospital, University Hospital Essen and one from the Department for Neurology—Institute of Translational Neurology, University Hospital Münster, Germany. All data concerning Patients 1 and 2 were extracted retrospectively from their medical files.

### 2.2. Antibody Analyses

Screening for myositis specific antibodies (Abs) was performed with line blot commercial immunoassays (Labor Berlin, Berlin, Labor Euroimmun, Luebeck, Germany) and included the Mi-2alpha und beta, TIF1g, MDA5, NXP2, Ku, PM-Scl 100/75, SRP, Jo-1, PL-7, PL-12, EJ, OJ, SAE, Ro-52. B, U1-RNP, Sm, SS-a/Ro-52, SS-B, Scl70, and CENP-B. Negative controls were used for the applied assay. Anti-HMGCR Ab was detected using a commercial Enzyme Linked Immunosorbent Assay (ELISA, Labor Volkmann, Karlsruhe, Germany).

### 2.3. Muscle Biopsy Investigations

Biopsies were obtained for diagnostic procedures including histology, enzyme histochemistry, immunofluorescence, and immunohistochemical investigations. Serial cryosections (10 μm) of transversely oriented muscle blocks were stained according to standard procedures with hematoxylin and eosin (H&E), Gömöri trichrome (GT), COX-SDH and SDH and nicotinamide adenine dinucleotide tetrazolium reductase (NADH-TR).

Immunofluorescence studies were performed using antibodies against α-Dystroglycan (α-DG) (Millipore #05-593, clone IIH6C4, 1:10), CD4 (Zytomed, clone SP35, ready-to-use), CD8 (DAKO, clone C8/144B, 1:100), CD68 (DAKO, clone EBM11, 1:100), MHC class I (DAKO, clone W6/32 1:1000), MHC class II (DAKO, clone CR3/43, 1:100), C5b-9 (DAKO/M777, clone aE11, 1:100) (data not shown). Immunofluorescence staining was performed in staining chambers after fixation in acetone for 10 min. The sections were then blocked with the appropriate serum (1:10 in PBS), dependent on the source of the secondary antibody, and incubated with the aforementioned primary antibodies over night at 4 °C or for 1 h at room temperature. After a washing step, the secondary antibody was added for 1 h. After a final washing step, the sections were aqueously mounted and stored at 4 °C.

Immunohistochemistry was conducted using antibodies against αB-crystallin (Abcam, ab13496, 1:2.500, mouse, clone 1B6.1-3G4), HSP70 (Abcam, ab6535, 1:100, mouse, clone BRM-22), LC3 (Nanotools Art, 0260-100, 1:50, mouse, clone LC3-2G6), LAMP2 (Santa Cruz Biotechnology, USA, SC-18822, 1:500, mouse, clone 5H2), and p62 (Abcam, ab91526, 1:100, rabbit, polyclonal). These immunoreactions were performed using the iVIEW-Ventana DAB (diaminobenzidine)-Detection Kit (Ventana, Tucson, AD, USA, 85755 USA). Appropriate biotinylated secondary antibodies were used, and visualization of the reaction product was carried out on a Benchmark XT immunostainer (Ventana) in a standardized manner. Cellular structures were counterstained with hematoxylin.

We used normal muscle tissue as a negative control (or physiological internal control, e.g., staining of major histocompatability class I (MHC class I) positivity of capillaries) for all reactions. Light microscopic investigations were performed using a Zeiss Axioplan epifluorescence microscope equipped with a Zeiss Axio Cam ICc1 and a Zeiss, BZ-X800 microscope (software: BZ-X800 Viewer).

## 3. Results

### 3.1. Clinical Presentations

#### 3.1.1. Patient 1

**Figure 1:**
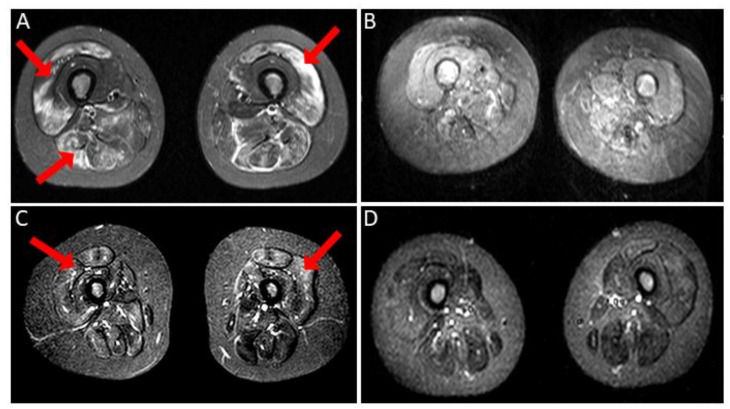
Magnetic resonance imaging (MRI) of both patients using the short tau inversion recovery (STIR) sequence. Patient 1: patchy, symmetrical edema of the thigh muscles at onset (**A**, arrows) and normalization of the STIR signal as well as marked fatty atrophy of the previously affected thigh muscles after one year of therapy (**B**). MRI–STIR sequence of Patient 2: patchy signal elevation predominantly in the quadriceps muscles, left more pronounced than the right, 4 months after starting therapy (**C**, arrows). Reduced signal elevations and atrophy of the quadriceps muscles at 10-year follow up (**D**).

The patient was born after an uneventful pregnancy, delivery and postnatal period were normal. She was age-adequately psychomotorically developed. At 8 years of age, signs of proximal muscular weakness occurred over a period of 8 months: her strength declined rapidly, and she was not able to climb stairs or to lift from the sitting position. Her maximum walking distance was 20 m. She developed dysphagia with swallowing difficulties and weight loss; restrictive pulmonary function with reduced coughing strength and forced vital capacity (FVC: 78%) were present. The childhood myositis assessment scale (CMAS) reflected her muscular weakness, with a score of 4/52 points. Laboratory findings showed raised CK of 10.710 U/L (50–240 U/L), LDH of 2.260 U/L (380–640 U/L), ASL of 336 U/L (<50 U/L), ALT of 310 U/L (10–45 U/L), and aldolase of 127 U/L (y7,6 U/L). CRP was negative. Cardial investigations (echocardiography, electrocardiogram) revealed normal results. In another clinic, due to lack of cutaneous signs for dermatomyositis, muscular dystrophy was first assumed, and investigation of a tailored genetic panel revealed no pathological mutation in the included genes (*ANO5, CAPN3, CAV3, DYSF, FKRP, GAA, MYOT, PYGM, SGCA, SGCB, SGCD, SGCG, TCAP*). No skin lesions were present at onset. Muscle magnetic resonance imaging (MRI) showed a symmetrical, patchy, elevated T2-weighted short tau inversion recovery (STIR) signal in the muscles of the pelvis, both thighs, and lower legs (Figure 1A).

**Figure 2:**
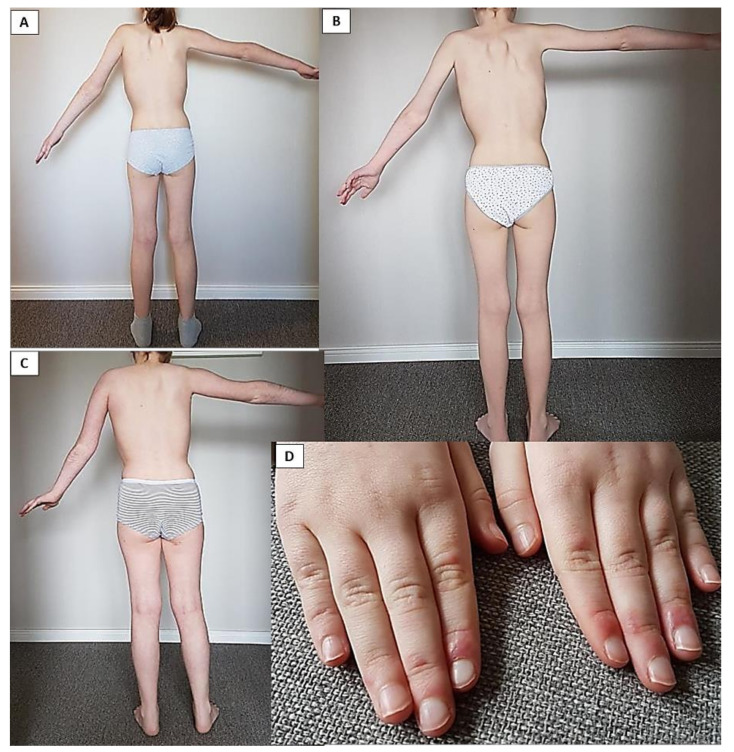
Patient 1 presenting asymmetrical weakness in her upper extremities. The patient presents the highest active elevation of her arms: (**A**): 3 months after methlyprednisolone intravenous therapy in combination with oral steroids and methotrexate; (**B**): 20-month follow up (rituximab, intravenous immunoglobulins, and methotrexate). The strength in her right arm was better compared to her left arm. Asymmetrical weakness persisted at the 2 years and 7 months follow up (**C**), and she developed skin lesions for the first time (periungual exanthema) (**D**).

**Figure 3:**
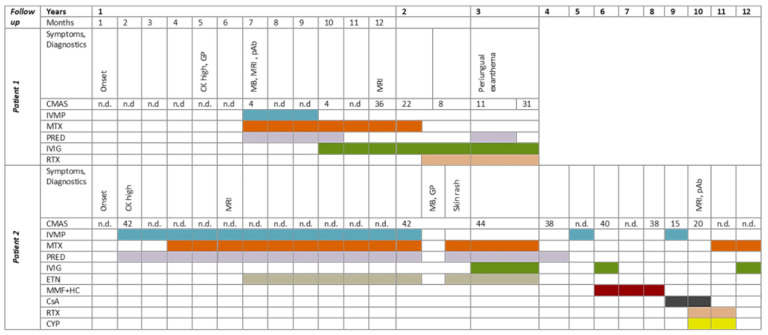
Timelines of both juvenile IMNM patients including onset of symptoms, diagnostics, and applied therapies. Patient 1 was followed for 3 years, and Patient 2 was followed for over 10 years. CMAS = childhood myositis assessment scale, maximal 52 points, CK = creatine kinase, GP = genetic panel, MB = muscular biopsy, MRI= magnetic resonance imaging, CsA = cyclosporine A, CYP = cyclophosphamide, ETN = etanercept, IVMP = intravenous methylprednisolone, MMF + HC = mycophenolate mofetile and hydroxychloroquine, MTX = methotrexate, PRED = prednisolone, RTX = rituximab, Onset = symptom onset, n.d. = not done, pAb = positive antibodies.

In myositis-panel analyses, positivity for SRP54-Ab was detected and therapy with pulsed methylprednisolone intravenous (IV) in combination with oral prednisolone and methotrexate was started. Under this therapy, her bulbar symptoms improved, but no improvement in her muscular strength was achieved although her CK levels decreased during the period of 3 months (1.639 U/L). Therefore, the therapy was switched to monthly IVIG (2 g/kg). Under this combination, her CK levels normalized, and her CMAS score improved to 36/52 12 months afterwards. She presented almost normal muscular strength in her lower extremities but developed persistent, asymmetrical weakness and atrophy in her upper extremities (Figure 2A–C). Her bulbar symptoms completely reversed, but her axial muscular weakness persisted. Her CK levels were within the normal range 15 months post-therapy start, and MRI follow up showed normalization of the T2 weighted STIR signal but revealed marked fatty atrophy of the previously affected muscles (Figure 1B). After two months, her CK-levels increased again (392 U/L), but her CMAS score remained stable. Due to the further increase in her CK levels and persistent weakness in her upper extremities as well as atrophy, therapy with rituximab was started. After two months, she developed severe weakness, her CK levels increased significantly (4.973 U/L), and her CMAS dropped to 8/52. Additionally, she developed a skin rash (Figure 2D), and oral steroid therapy was re-started. With this combination, she improved again, achieving a CMAS score of 26/52. She received rituximab three times (375 mg/m^2^), and within 7 months, a normalization of her CK levels was achieved. Due to a lack of improvement, methotrexate-treatment was stopped, and she remained on therapy with IVIG. Her CK levels rose again, and a fourth dose of rituximab was applied (Figure 3). Her CMAS score remained at 29/52, and her CK levels normalized. At the end of follow up, she was able to walk a distance of over 1000 m, but she had to hold onto a railing when climbing stairs and had positive Gower’s phenomena when rising up from the floor, but she was able to stand up from the sitting position unsupported.

#### 3.1.2. Patient 2

The mother´s pregnancy and birth of the female patient were uneventful. She showed age-adequate psychomotor development. At the age of 9 years, signs of proximal muscular weakness occurred, and over the period of 1 month her muscle strength declined rapidly and she presented with increasing difficulties when climbing stairs. Her CMAS score was 42/52 points. She had proximal muscle weakness and had to hold onto railing when climbing stairs. In addition, she suffered from high fever for several days before her first hospital admission. An MRI of the thighs after 4 months of therapy showed patchy signal enhancement predominantly in both quadriceps muscles (left > right, Figure 1C). Laboratory findings showed raised serum CK of 18.000 U/L (50–240 U/L), LDH of 1.862 U/L (380–640 U/L), AST of 532 U/L (<50 U/L), and ALT of 900 U/L (10–45 U/L). CRP was negative. Cardial investigations (echocardiography, electrocardiogram) revealed normal results. Moreover, skin exanthema appeared in the neck region. A biopsy of the skin revealed suspected interstitial granulomatous dermatitis without typical findings for dermatomyositis.

**Figure 4:**
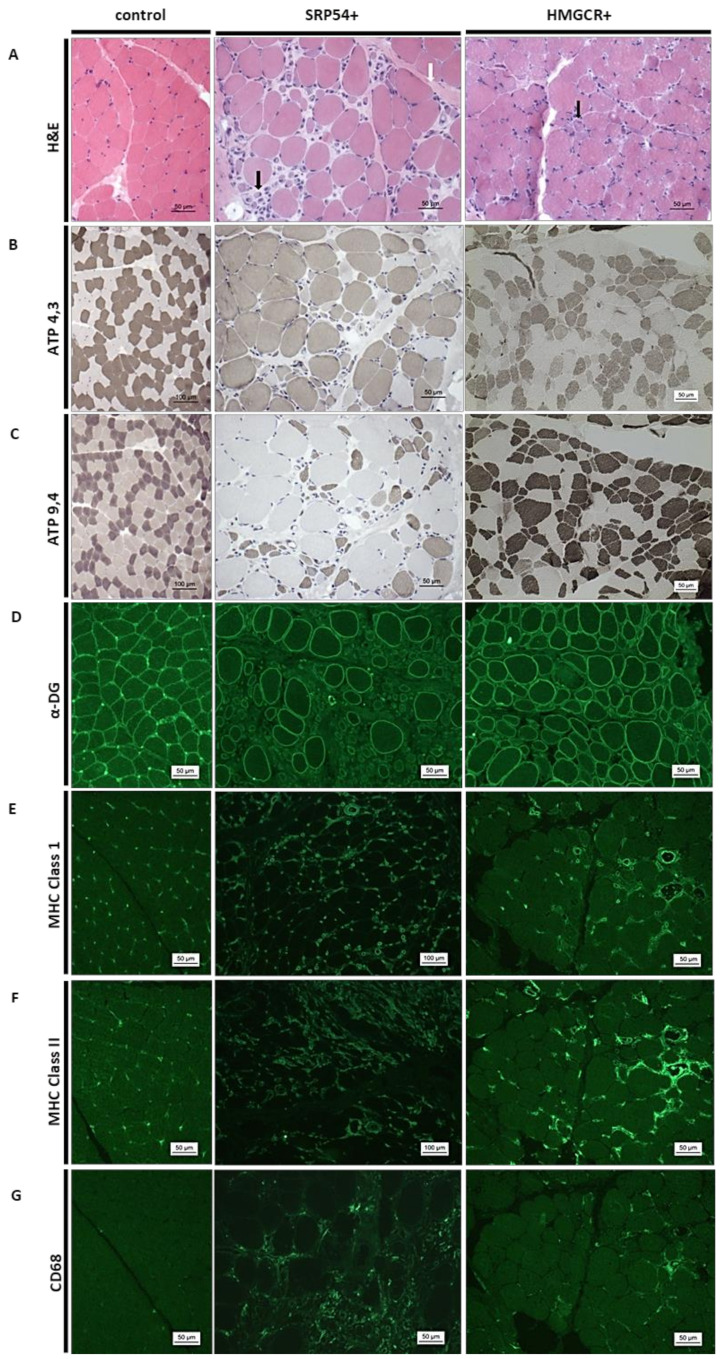
Muscle biopsy findings in pediatric IMNM patients; the middle column seropositive patient for SRP54, and the right column is seropositive patient for HMGCR. Patient 1 (SRP54+): perimysial proliferation of connective tissue (**A**, white arrow) is detectable but no lymphohistiocytic cell infiltrates are observed. Moreover, marked fibre size variability with numerous atrophic fibres are present (**A**, black arrow). Increased type 1 fibres and predominantly atrophic type-2 fibres (**B**,**C**) are identified by ATPase staining. Immunofluorescence studies revealed α-dystroglycan-positive and α-dystroglycan-negative necrotic fibres (**D**). Moreover, major histocompatibility class I (MHC class I) immunoreactivity is increased in degenerating fibers in addition to a non-specific reactivity at the sarcolemma and within the sarcoplasm of many fibres (**E**). Along this line, MHC II is markedly increased at the vessels, subsarcolemally, and in necrotic fibres (**F**). Foci of regeneration are associated with small basophilic fibres and with large nuclei clusters of regenerating fibres that are also admixed with many macrophages (CD68-positive) (**G**). Patient 2 (HMGCR+): H&E staining revealed marked fibre size variability and hypertrophy, grouped atrophic fibers (black arrow), diffuse cell necrosis, and phagocytosis as well as basophilic regenerating fibres often clustering in groups (**A**). Immunofluorescence studies of the α-dystroglycan protein fragments showed a mosaic pattern of α-dystroglycan-positive and α-dystroglycan-negative fibres (**D**). MHC class I and II upregulation is present perivascular and only rarely present subsarcolemally (**E**,**F**). Increased CD68 expression is detectable in the perivascular region and in necrotic fibres (**G**).

Under the suspicion of a JDM, treatment was initiated with pulsed methylprednisolone IV (20 mg/kg/day on three consecutive days) every 2nd week for 3 months and once/month afterwards. Additionally, she received 5 mg oral prednisolone in combination with methotrexate (15 mg/m^2^ weekly) (Figure 3). After six months, etanercept (25 mg/day) was added due to persistent clinical symptoms with proximal limb weakness, highly elevated serum CK (3.932 U/L), and radiological signs of inflammation in both quadriceps muscles. Her muscular strength improved over the 12-month period, but she developed reduced muscular endurance (CMAS 44/52). Immunosuppressive therapy was stopped due to negative screening for myositis-specific Abs (HMGCR Ab was not included in the panel), and muscular dystrophy was assumed. A muscle biopsy showed myopathic features, and staining of α-dystrogycan protein fragments showed a mosaic pattern of α-dystrogycan-positive and α-dystrogylcan-negative fibres with scattered lympho-monocytic infiltrates (Figure 4D). Commercial genetic panel testing for common dystroglycanopathy genes was negative (*FKRP, FKTN, POMT1, ISDP, DYSF*).

After another two months, she developed a typical skin rash (on her finger extensor regions and on her trunk), and immunosuppression was restarted (prednisolone, methotrexate and etanercept) (Figure 3). Unfortunately, the patient showed signs of steroid induced osteonecrosis on both femurs, so therapy with pulsed IV methylprednisolone was discontinued, and therapy with IVIG was given over period of 9 months (2 g/kg every month). With this treatment regime, muscle strength improved significantly in both limbs. However, as relevant deficits persisted (CMAS 40/52), treatment with methotrexate and etanercept was discontinued, and therapy with mycophenolate mofetil (2 × 500 mg/day) was initiated in combination with hydroxychloroquine, followed by another treatment switch after one year (cyclosporine A). Her CK levels remained high (2.773 U/L). After a clinically stable period of 2 years, a rapid clinical deterioration (CMAS 15/52) was observed. Therefore, she received additional steroid pulse therapy every month for half a year without relevant clinical improvement. Further diagnostics were initiated with screening for further myositis-specific Abs, including HMGCR-Ab for the first time. The latter was repeatedly highly elevated >200 U/mL (<20 U/mL). MRI control (10 years after the first symptoms) showed atrophy in both quadriceps muscles with only sparse inflammation (Figure 1D). HMGCR-Ab positive IMNM was diagnosed, and the patient received rituximab as monotherapy, resulting in clinical stabilization after three cycles (CMAS 19/52). Unfortunately, 3 months after the last cycle, a rapid decrease of muscle strength in both legs was documented, resulting in a further treatment switch to IV cyclophosphamide (350–500 mg/m^2^) every month. Although disease stability was achieved, therapy with cyclophosphamide was stopped after 19 cycles due to persistent lymphopenia. Again, a treatment regime with methotrexate and IVIG was initiated after the lymphocyte normalization (Figure 3).

### 3.2. Muscle Biopsy Findings

To assess the pathology of skeletal muscle in pediatric IMNM patients, we examined general histological alterations in the biopsies of two patients. Small, mostly rounded fibres were identified accompanied by increased variation in fibre size and increased fibrosis in the endomysium as well as enlargement of the perimysium (on H&E, Figure 4A). Foci of regeneration with small basophilic fibres with large nuclei clusters of regenerating fibres were admixed with many macrophages and some lymphocytes in Patient 1 (Figure 4A, SRP+). Additionally, we identified that the staining of α-dystroglycan protein fragments showed a mosaic pattern of α-dystroglycan-positive and α-dystroglycan-negative fibres in Patient 2 (Figure 4D, HMGC+). Major histocompatibility class I (MHC class I) immunoreactivity was increased in the degenerating fibres and nonspecifically on the sarcolemma and the sarcoplasm of many fibres (Figure 4E). In addition, MHC II immunoreactivity was increased at the vessels, subsarcolemally, and in necrotic fibres (Figure 4F). Regeneration foci with small basophilic fibres with large nuclei clusters of regenerating fibres were admixed with many macrophages (Figure 4A,G).

**Figure 5:**
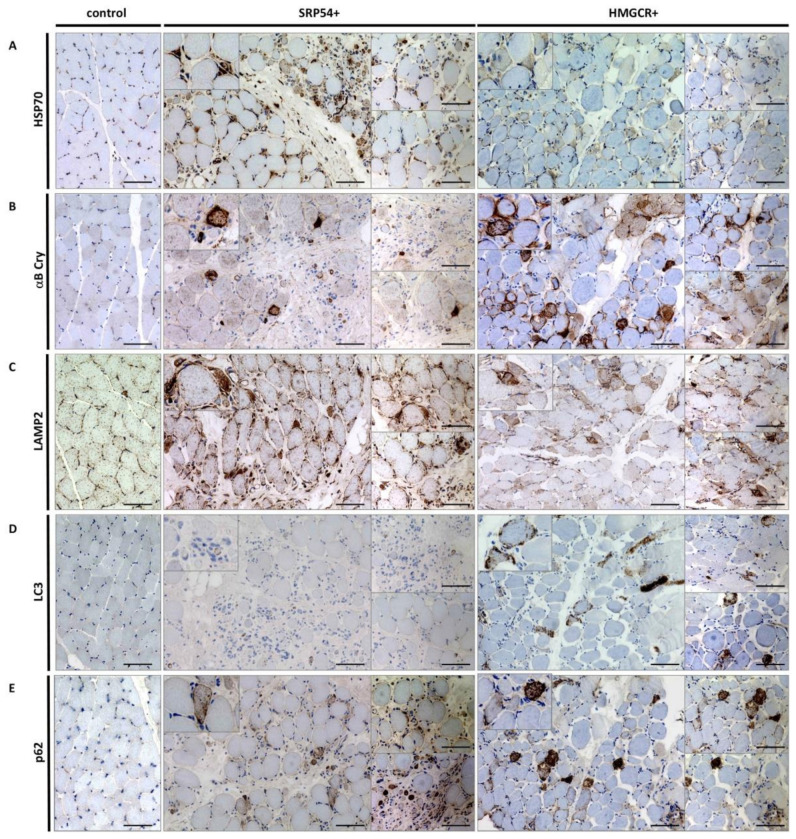
Histological findings in both juvenile IMNM patients with focus on the protein clearance machinery. Histological staining of markers involved in proteostasis showed upregulation of all investigated proteins in both juvenile IMNM patients, with subtle differences in staining intensity. (**A**): HSP70 and (**B**): αB-crystallin show clear upregulation in both patients as well as (**C**): LAMP2, which is stained diffusely on the sarcoplasm. (**D**): LC3 shows a fine granular pattern and is stronger in the HMGCR+ patient, while (**E**): p62 is intense in both patients. A typical pattern in IMNM patients is seen. Necrotic muscle fibres stain is unspecific for all markers. Scale bar = 100 µm.

To investigate if juvenile IMNM patients share the pathophysiological cascades that are known to take place in adult cases, we next focused on proteostasis by immunological examination of biochemical markers including chaperones, protein clearance proteins, and a protein aggregation marker known to be increased in adult IMNM patients [11]. We therefore chose some markers that are crucially involved in the chaperone-assisted selective autophagy (CASA) pathway. Staining of HSP70 and αB-crystallin demonstrated clear upregulation in both patients, whereby the intensity of HSP70 staining in the HMGCR patient was less strong (Figure 5A,B). Lysosomal staining with LAMP2 additionally revealed diffuse sarcoplasmic stains with the same intensity in both patients, showing high autophagy activity. No clustering in the perifascicular regions or specific parts of the fascicle could be seen (Figure 5C).

As previously shown in adult patients [12], the staining pattern of LC3+ and p62+ muscle fibres show a fine granular pattern throughout the entire sarcoplasm, which is common for IMNM patients and can also be seen in both juvenile patients (Figure 5D,E). Especially in the SRP54+ patient, numerous fibres are intensely immunoreactive for p62.

## 4. Discussion

IMNM in children is a rare but is relevant differential diagnosis to juvenile dermatomyositis and presents a diagnostic challenge due to mostly lacking typical cutaneous manifestations and only partial response to first line immunosuppressive therapy with steroids and methotrexate. The increased level of CK values or typical findings indicative for myositis on the MRI level do not always occur simultaneously in combination with clinical aggravation in our patients; this, in turn, makes the decision when to change or escalate therapy challenging.

### 4.1. Comparison to Previously Reported Juvenile and Adult IMNM Cases

In adult patients with anti-SRP54 and anti-HMGCR positive Abs, acute-to-subacute, symmetrical progressive proximal weakness is present in both subgroups and muscular weakness affects the legs more than the arms [6]. However, anti-SRP54 Ab positive patients have more neck weakness and dysphagia compared to HMGCR-Ab patients. Consistent with this, Patient 1 in our study presented with bulbar symptoms at disease onset. Muscular atrophy was significantly more present in SRP54-seropositive cases compared to HMGCR-positive patients, although it seems that a longer active disease period also leads to muscular atrophy in the latter group [6,10]. Of note, younger adult patients had a more severe disease course accompanied by a worse prognosis compared to older patients in long-term follow-up studies [6,15]. A similar presentation as in adults, but with additional distal weakness, was also reported in a small population of pediatric patients with SRP54- and HMGCR-Ab positive serostatus. Interestingly, muscular atrophy in combination with mild to moderate muscular weakness despite intensive therapy was present in 86–100%, which is higher compared to other myositis-specific Abs [5]. This clinical aspect could also be observed in our patients, who both presented muscular atrophy at the last follow up, and, in the case of the anti-SRP54 positive patient, already early in the course of the disease. The anti-SRP54 positive patient also had asymmetrical muscle weakness, which so far has not been reported as a specific symptom, and, in case of our patient, asymmetric weakness remained despite intensive therapy during long-term follow up (Figure 2A–C).

### 4.2. Microscopic Findings

In both patients, the presence of a genetically based muscular dystrophy was suspected at the beginning or during the course of the disease. This suspicion arose due to the unsatisfactory response to immunosuppressive therapy and a lack of specific Abs in the first testing period (Patient 2) and the clinical presentation with absent dermatomyositis-like cutaneous symptoms (both patients) at onset as well as based on the asymmetric muscle weakness (Patient 1). In the group with mostly adult IMNM-patients, necrosis and regeneration of muscle fibres and endomysial fibrosis with no or little endomysial lymphocyte infiltration was present [6,10]. This pathomorphological observation was also present in 2/3 juvenile SRP54-Ab positive patients, comparable with the findings in Patient 1 in our study (Figure 4A). In one patient, inflammatory infiltrates were additionally present, implicating an autoimmune disorder [16]. Moreover, in Patient 2, muscular dystrophy was assumed based on the histologic findings and α-dystrogycan-negative fibres. The misdirection toward a limb-girdle muscular dystrophy (LGMD) has been described previously [10]. In a small group of six HMGCR-Ab positive patients with a longer period of disease duration (3.5 to 23 years), muscle histology showed chronic myopathic features, such as myofibre atrophy, fibre size variability, splitting myofibres, and increased endomysial fibrosis. However, no signs of primary inflammation were present [10]. Similar as in our patient, all patients underwent prior to diagnosis genetic testing for common LGMDs, and in two patients, facioscapulohumeral dystrophy was assumed due to asymmetrical weakness [10].

The histological and immunohistochemistry findings in Patient 1 are comparable to those seen in adults with IMNM. As in adults, the focus is not on the inflammatory process with endomysial lymphocytic inflammation surrounding non-necrotic myofibre, as is the case in JDM, but on fibrous necrosis and macrophage activation with sparse inflammatory infiltrates [6,8,13]. In the Patient 2, muscle biopsy presented more of a myopathic picture, which is not primarily related to IMNM, but has rarely been described in juvenile and adult patients with positive for HMGCR [10,17,18].

Immunostaining studies focusing on proteins known to modulate protein clearance and to be upregulated in adult IMNM patients [11] revealed an increase of sarcoplasmic chaperones (HSP70 and αB-crystallin), modulators of autophagy (LAMP2 and LC3), and a protein aggregation marker (p62), thus also indicating perturbed proteostasis in the disease cause of juvenile patients and along this line confirm the pathophysiological findings described by Preuße and co-workers [11,12]. Given that the modulation of proteostasis is a well-known therapeutic target in a variety of neuromuscular disorders (e.g., [19,20]), by applying chemical chaperones and/or autophagy inducers, our finding might open new avenues for additional therapeutic concepts. In this context it is important to note that several of these drugs are already FDA approved.

### 4.3. Therapeutic Regimen/Outcome

Taken together, data reported in the literature and our own clinical observations indicate that anti-HMGCR Ab patients tend to have a less progressive disease course and better response to therapy: anti-HMGCR patients responded better to steroid therapy compared to anti-SRP54-seropositive patients, with a higher frequency of steroid monotherapy in the anti-HMGCR group [6]. In both groups, therapy with IVIG is recommended in case of a failed response to steroids or a severe disease course [13]. An early intensive therapy with IVIG, methotrexate, and rituximab and/or cyclosphosphamide prevented further progression and even improved clinical symptoms in a small pediatric case series of three patients with anti-SRP54 Ab and a follow-up period of 20–50 weeks [16]. Both of our patients only showed partial and unsatisfactory response to pulse methylprednisolone therapy in combination with methotrexate or etanercept. Under IVIG-treatment, both achieved a longer period of stability (Patient 1 one year, Patient 2 two years in combination with additional immunosuppression), but this was not effective in the long term. Patient 1 showed a renewed increase in CK levels without simultaneous worsening of the CMAS score; this raises the question of reacting to rising CK values in these patients by intensifying the therapy before the clinical symptoms worsen. We also assume that a longer follow-up period is needed for a final assessment of the influence of the therapy due to the undulating course of the disease.

In patients with HMGCR Abs, however, there seems to be a small group that shows little clinical activity over a longer period of time; here, the extent of muscle atrophy seems to be an important parameter to predict the response to therapy [10]. Remarkably, this could be observed also in our patient with a steady state under IVIG therapy for two years, but the further progression of muscular weakness with the development of atrophy and only a partial response to further escalation with rituximab and cyclophosphamide.

Interestingly, in Patient 2, muscular symptoms worsened after respiratory infection with fever, implicating some role of additional inflammation as a possible disease activating factor. In reported SRP54-positive cases, infection or coryzal illness preceded in more than half of the cases at the onset of muscular weakness [16,21,22,23].

## 5. Conclusions

In children with a new onset of symmetrical or asymmetrical muscle weakness without cutaneous features associated with dermatomyositis, remarkably high CK-levels, and previously inconspicuous psychomotor development, the presence of inflammatory myopathy should be considered since the appropriate therapeutic options exist in contrast to genetically determined muscular dystrophies. Anti-HMGCR Ab is not always included in the commercial myositis panel, and this possibility must be considered in combination with specific clinical, MRI, and muscle biopsy findings. We believe that in the case of IMNM, clinical presentation in combination with the detection of specific antibodies, MRI changes and muscle biopsy will allow a correct and rapid diagnosis that allows an early start of therapy. In this population, an early and intensive therapy may be crucial for outcome in long-term. Patients should be followed for a long period of time due to the undulating course of the disease, and the worsening of muscular strength and persistently high CK-levels should implicate early escalation or re-start of the immunosuppression. Consistent with adult IMNM, juvenile IMNM-patients also present with perturbed proteostasis, a biochemical observation that might open new avenues for the application of novel therapeutic concepts in the future.

## Data Availability

The data that support the findings of this study are available from the corresponding author upon reasonable request.
